# Current News

**Published:** 1905-09

**Authors:** 


### Current News.

### FIRST ANNUAL CLINIC OF THE FRATERNAL DENTAL SOCIETY OF ST. LOUIS.

THE first annual clinic will be held November 20 and 21, 1905, at the Barnes Dental College. Special features of the meetings will be a series of lectures on "Cavity Preparation," "Methods and Principles of packing Gold," and "Methods and Principles of finishing Fillings," by Dr. E. K. Wedelstaedt, of St. Paul; Drs. A. C. Searl, of Owatonna, Minn., J. F. Wallace, of Canton, Mo., and numerous other members of the Black and Wedelstaedt Clubs, and other prominent men in Operative and Prosthetic Dentistry will give clinics. Complete programme will be announced later.

All ethical practitioners are invited to be present and clinic. Please send your name and subject of clinic to the secretary. Exhibit space to be obtained by application to the secretary.

A cordial invitation is extended to the profession to be present and make this meeting limited in scope but limitless in importance, the best ever held in this section.

BURTON LEE THORPE, President.
S. H. VOYLES, Secretary,
3201 Washington Avenue.

